# Hypercalcemia in metastatic GIST caused by systemic elevated calcitriol: a case report and review of the literature

**DOI:** 10.1186/s12885-015-1823-7

**Published:** 2015-10-24

**Authors:** Katrine Hygum, Christian Nielsen Wulff, Torben Harsløf, Anders Kindberg Boysen, Philip Blach Rossen, Bente Lomholt Langdahl, Akmal Ahmed Safwat

**Affiliations:** 1 Department of Endocrinology and Internal Medicine, Aarhus University Hospital, Tage-Hansens Gade 2, DK-8000 Aarhus, C Denmark; 2 Department of Oncology, Aarhus University Hospital, Nørrebrogade 44, DK-8000 Aarhus, C Denmark

**Keywords:** Hypercalcemia, Systemic hypervitaminosis D, Calcitriol, GIST

## Abstract

**Background:**

Hypercalcemia is the most common oncologic metabolic emergency but very rarely observed in patients with gastrointestinal stromal tumour, which is a rare mesenchymal malignancy of the gastrointestinal tract. We describe a case of hypercalcemia caused by elevated levels of activated vitamin D in a patient with gastrointestinal tumour. Prior to this case report, only one paper has reported an association between hypercalcemia, gastrointestinal stromal tumours and elevated levels of vitamin D.

**Case presentation:**

An otherwise healthy 70-year-old Caucasian woman, previously treated for duodenal gastrointestinal stromal tumour, was diagnosed with liver metastasis, and relapse of gastrointestinal stromal tumour was confirmed by biopsy. At presentation, the patient suffered from severe symptoms of hypercalcemia. The most common causes of hypercalcemia, hyperparathyrodism, parathyroid hormone-related peptide secretion from tumour cells, and metastatic bone disease, were all dismissed as the etiology. Analysis of vitamin D subtypes revealed normal levels of both 25-OH Vitamin D2 and 25-OH Vitamin D3, whereas the level of activated vitamin D, 1,25 OH Vitamin D3, also referred to as calcitriol, was elevated.

**Conclusion:**

The fact that plasma calcitriol decreased after initiation of oncological treatment and the finding that hypercalcemia did not recur during treatment support the conclusion that elevated calcitriol was a consequence of the gastrointestinal stromal tumour. We suggest that gastrointestinal stromal tumours should be added to the list of causes of humoral hypercalcemia in malignancy, and propose that gastrointestinal stromal tumour tissue may have high activity of the specific enzyme 1α-hydroxylase, which can lead to increased levels of calcitriol and secondarily hypercalcemia.

## Background

Hypercalcemia is the most common oncologic metabolic emergency. Up to 30 % of all cancer patients will experience tumour-induced hypercalcemia (TIH) [1, 2]. The most common reason is humoral hypercalcemia of malignancy (HHM) which is caused by parathyroid hormone-related peptide (PTHrP) secretion from tumour cells (approximately 80 % of cases), followed by metastatic bone disease (approximately 20 %). In a few percent of cases, hypercalcemia is caused by tumour cells producing 1,25 OH-Vitamin D or parathyroid hormone (PTH) [1].

The malignancies most often associated with hypercalcemia are multiple myeloma, breast, lung, and renal cell carcinoma [1]. Patients suffering from gastrointestinal stromal tumour (GIST) very rarely experience hypercalcemia [3].

GIST is a rare neoplasm but remains the most common mesenchymal tumour of the gastrointestinal tract, with an incidence of 11–19.6 per million and a median age of diagnosis around 65 years. Most often, the tumour is localized at presentation, but up to half of the patients will suffer from recurrence, which most frequently occurs in the peritoneal cavity or in the liver. GIST is highly resistant to conventional chemotherapy, however, following the introduction of tyrosine kinase inhibitors, e.g. imatinib, in both the preoperative, adjuvant and metastatic setting the prognosis has dramatically improved [4].

Below, we present a case of hypercalcemia in a patient with recurrent GIST disease.

## Case presentation

On June 12 2014, a 70-year-old Caucasian woman was referred to the fast track cancer referral programme at Aarhus University Hospital, Denmark, due to a palpable abdominal mass, a 4–5 kg weight loss, and fatigue. Six years previously the patient had undergone a Whipple operation where a 3.8 cm duodenal GIST tumour was radically resected. After surgery the patient was followed with regular CT scans for four and a half years. No adjuvant treatment with imatinib was given, as this was not standard of care for high risk patients in Denmark in 2008. After the cessation of her follow-up, the patient had been well until her weight loss in spring 2014.

Initial routine biochemistry revealed elevated p-ionized calcium; 2.11 mmol/L, suppressed p-PTH; 1.5 pmol/L, and impaired renal function with an estimated glomerular filtration rate (eGFR) of 37 mL/min, one year previously eGFR was 88 mL/min. Finally, p-total alkaline phosphatase was slightly elevated to 148 U/L but other routine biochemistry was normal. At the time of surgery in 2008 p-total calcium was not elevated, and p-ionized calcium was not measured.

To further characterize the cause of hypercalcemia, plasma levels of monoclonal protein, calcitriol and PTHrP were measured. Monoclonal protein and PTHrP were undetectable but calcitriol was more than twofold elevated (375 pmol/L). See Table 1 for biochemistry.

**Table 1:** Blood analyses

	Normal range	July 20, 2012	June 12, 2014	June 13, 2014	June 14, 2014	June 15, 2014	June 15, 2014	June 18, 2014	June 20, 2014	June 27, 2014	July 04, 2014	October 17, 2014
Creatininium	45–90 mikromol/L	59	**123**	**108**	**95**	**99**	**93**	82	83	82	67	62
Calcium (ion)	1.18–1.32 mmol/L	-	**2.11**	**1.71**	**1.82**	**2.17**	**1.74**	**1.60**	**1.61**	1.29	-	1.29
Alkaline phosphatise	35–105 U/L	101	**148**	-	-			-	**127**	-	100	**132**
PTH	1.6–6.9 pmol/L	6.1	**1.5**	-	-			-	-	-	6.3	3.3
PTHrP	<2.6 pmol/L	-	-	-	-			-	<1.9	-	-	-
D2 (25-OH Vitamin D2)		<10	<10	-	-			-	-	-	-	<10
D3 (25-OH Vitamin D3)		78	81	-	-			-	-	-	-	117
Calcitriol (1,25 (OH)2 vitamin D3)	60–180 pmol/L	-	**375**	-	-			-	-	-	**224**	**181**

Values outside the local reference range are shown in bold

Plasma levels of sodium, potassium, magnesium, and TSH were normal at all timepoints

A regular CT scan was followed by a PET-CT scan and two hepatic tumours, measuring 11.0 × 8.6 cm and 9.2 × 8.2 cm were found, but no bone lesions. Ultra-sound guided biopsy from one of the hepatic tumours concluded recurrence of GIST with the following morphological and immunohistochemical characteristics: Two mitoses per 8 HPF; Ki-67 was 5 % on average but up to 20 % in hot spots; mutation in KIT exon 9 but no mutations in PDGFRA Exon 18.

**Fig. 1:**
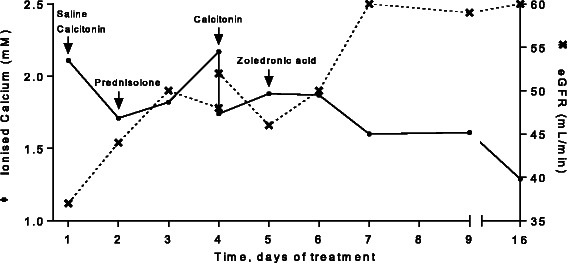
Changes in plasma-ionized calcium and estimated glomerular filtration rate (eGFR) in response to treatment. Treatment administered: intravenous saline (day one), 600 IU of calcitonin (days one and four), 37.5 mg/day of prednisolone (day two and onwards), and 4 mg of intravenous zoledronic acid (day five)

The hypercalcemia was initially treated with intravenous saline and 600 IU of calcitonin and the day after oral prednisolone 37.5 mg/day was initiated. P-ionized calcium initially decreased to 1.71 mmol/L but on day four increased again to 2.17 mmol/L and another dose of calcitonin was administered. The results of the p-25-hydroxy vitamin D analyses appeared on day five and as expected, 25-OH Vitamin D2 was unmeasurable and 25-hydroxy vitamin D3 normal (81 nmol/L). Hence, as the risk of bisphosphonate-induced hypocalcemia was thought to be minimal, intravenous zoledronic acid (4 mg) was administered (Fig. 1).

The patient was discharged on day 7 with the recommendation of increased oral fluid intake.

Oral imatinib therapy started on day 10 at the oncology department, and the patient was followed up by regular controls. Plasma level of ionized calcium was normalized on day 16 and prednisolone was tapered off and ultimatively stopped on day 33. There were no adverse or unanticipated events related to the treatment.

Two weeks after initiation of imatinib, p-calcitriol had decreased to 224 pmol/L. A follow-up CT scan eight weeks after the first showed a significant reduction in tumour size. Imatinib was continued and after 15 weeks of treatment p-calcitriol was nearly normalized at 181 pmol/L. P-ionized calcium remained in the reference interval.

## Discussion

Various vitamin D molecules (calciferols) exist, the two of principal importance being vitamin D3 and Vitamin D2. The major source of vitamin D is dermal synthesis of Vitamin D3, cholecalciferol, when exposed to ultraviolet light. Vitamin D3 also occurs in food and can be taken as a supplementary vitamin. In the non-hydroxylated form vitamin D3 is metabolically inactive. The first step towards activation takes place in the liver and is hydroxylation at position 25, this is followed by hydroxylation at position 1, which primarily takes place in the kidneys and is mediated by the enzyme 1α-hydroxylase. The activated vitamin D; 1,25-dihydroxy-vitamin D3 (=1,25-OH vitamin D3) is also called calcitriol [5].

**Fig. 2:**
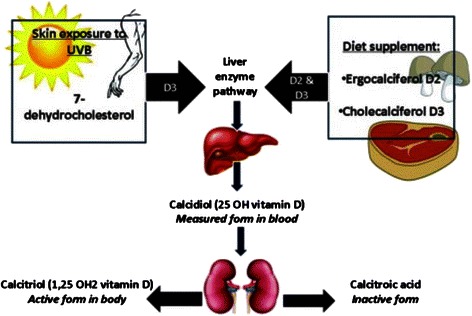
Vitamin D synthesis. Taken from Mostafa et Hegazy [22]. Synthesis and activation of Vitamin D subtypes

Vitamin D2, ergocalciferol, (produced by some fungi) is of minor importance in humans if not consumed as a vitamin supplement. Figure 2 shows the synthesis of active vitamin D.

Both monohydroxylated vitamin D and dihydroxylated vitamin D bind to the vitamin D receptor (VDR) in the small intestines, kidneys and bones, but calcitriol binds with a much stronger affinity than the other metabolites [6]. Stimulation of the VDRs increases intestinal absorption and renal reabsorption of calcium and promotes mineralization of bone [7].

Most cases of TIH are caused by either HHM or osteolytic bone metastasis. In this patient, alkaline phosphatase was elevated but the patient was found to be without bone metastasis. HHM was dismissed by a negative blood test for PTHrP, and hyperparathyrodism from ectopic PTH production, a rare cause of hypercalcemia in cancer, was dismissed by decreased plasma PTH. In some cancers including multiple myeloma, breast cancer, prostate cancer, and renal carcinoma hypercalcemia may be induced by production of osteoclast-activating factors such as tumor necrosis factor alpha, interleukine 6, and receptor activator of nuclear factor-κB ligand (RANKL) [8, 9]. In our case, however, both the PET-CT and the absence of monoclonal protein in the blood ruled out these cancers.

Analysis of vitamin D subtypes revealed normal levels of both 25-OH Vitamin D2 and 25-OH Vitamin D3, whereas 1,25 OH Vitamin D3 (calcitriol) was elevated. Reasons for a picture with elevated calcitriol, normal 25-OH vitamin D, and minimally lowered PTH could be treatment with calcitriol (sometimes used in the treatment of parathyroid or kidney disease) or endogenous production of calcitriol. The patient, however, was not treated with calcitriol.

Extra-renal 1α-hydroxylase activity and autocrine/paracrine secretion of calcitriol have been detected in normal tissue (skin, breast, immune system, bone, and intestines) although activity of the enzyme is not sufficient to elevate calcitriol in the blood [6, 10].

Recently, most types of cancer tissue have been found to express autocrine/paracrine 1α-hydroxylase activity but it is not clear whether the aberrant regulation of the vitamin D system is a consequence of the malignant transformation or contributes to tumour development [11].

It is well-known that hypercalcemia caused by extra-renal 1α-hydroxylase activity may occur in patients suffering from lymphoma [12] and in patients with benign granulomatous diseases such as sarcoidosis [13]. Based on the clinical picture and laboratory investigations our patient was judged free from these diseases.

Elevated calcitriol as a stand-alone biochemical cause of hypercalcemia has only been reported in a few patients with solid cancers (other than lymphomas) [14–17]. Evans et al. analysed the expression of 1α-hydroxylase in tissue from 12 patients with dysgerminomas. They concluded that the enzyme was expressed by both tumour cells and macrophages associated with the tumour; the localised produced calcitriol eventually spilled over into the circulation causing hypercalcemia. The authors speculated if the high expression of 1α-hydroxylase in the tumour is part of the immune response [15].

In the present case, the fact that the hypercalcemia did not respond to treatment with inhibition of osteoclast activity by intravenous zoledronic acid, also indicates a non-bone related mechanism of TIH. In general glucocorticoid therapy is expected to lower the calcium levels within three to five days and it has previously been shown that glucocorticoids are specifically effective when treating calcitriol-induced hypercalcemia [18]. Hence, the insufficient response to oral glucocorticoid therapy five days after initiation is a surprising finding which could be caused by an insufficient oral dose of glucocorticoid. The conclusive treatment of the hypercalcemia appeared to be the tyrosine kinase inhibitor imatinib, which has previously proved effective in other cases of TIH in GIST [3, 19, 20]. One could speculate whether treatment with tyrosine kinase inhibitors is generally effective in critical TIH and should be tried when the standard treatment of hypercalcemia fails.

Regarding GIST, we have found only four reports on hypercalcemia. In two of the cases, the reason for hypercalcemia was not sought [19, 21], in one case hypercalcemia was found to be PTHrP-mediated [20], and in the last case hypercalcemia was found to be calcitriol-mediated (not secondary to elevated PTH or PTHrP) [3].

In our case, elevated calcitriol was the only identified reason for the hypercalcemia. We believe that GIST tumour cells or tumour-associated cells possessed 1α-hydroxylase activity causing elevated p-calcitriol and p-ionized calcium.

## Conclusions

In conclusion, hypercalcemia in a patient with GIST is a very rare phenomenon; moreover, this is only the second case report proposing that GIST tissue have high 1α-hydroxylase activity which can lead to increased levels of calcitriol and secondarily hypercalcemia. Our finding is corroborated by the fact that oncologic treatment lead to a reduction in tumor size with contemporary decreasing levels of p-calcitriol. To validate this finding, we recommend analysis of 1α-hydroxylase activity in GIST tissue especially from patients presenting with hypercalcemia and elevated calcitriol.

## Consent

Written informed consent was obtained from the patient for publication of this case report and any accompanying images. A copy of the written consent is available for review by the Editor of this journal.

## Abbreviations

CT: Computed tomography
eGFR: Estimated glomerular filtration rate
GIST: Gastrointestinal stromal tumour
HHM: Humoral hypercalcemia of malignancy
HPF: High power field
p-: Plasma
PET-CT: Positron emission tomography-computed tomography
PTH: Parathyroid hormone
PTHrP: Parathyroid hormone-related peptide
TIH: Tumour-induced hypercalcemia
TSH: Thyroid-stimulating hormone
VDR: Vitamin D receptor
